# Exposure and infection to *Plasmodium knowlesi* in case study communities in Northern Sabah, Malaysia and Palawan, The Philippines

**DOI:** 10.1371/journal.pntd.0006432

**Published:** 2018-06-14

**Authors:** Kimberly M. Fornace, Lou S. Herman, Tommy R. Abidin, Tock Hing Chua, Sylvia Daim, Pauline J. Lorenzo, Lynn Grignard, Nor Afizah Nuin, Lau Tiek Ying, Matthew J. Grigg, Timothy William, Fe Espino, Jonathan Cox, Kevin K. A. Tetteh, Chris J. Drakeley

**Affiliations:** 1 Faculty of Infectious and Tropical Diseases, London School of Hygiene and Tropical Medicine, London, United Kingdom; 2 Infectious Diseases Society Kota Kinabalu- Menzies School of Health Research Clinical Research Unit, Kota Kinabalu, Malaysia; 3 Faculty of Medicine and Health Sciences, Universiti Malaysia Sabah, Kota Kinabalu, Malaysia; 4 Research Institute of Tropical Medicine, Department of Health, Muntinlupa City, Philippines; 5 Biotechnology Research Institute, Universiti Malaysia Sabah, Kota Kinabalu, Malaysia; 6 Menzies School of Health Research and Charles Darwin University, Darwin, Australia; 7 Jesselton Medical Centre, Kota Kinabalu, Malaysia; Walter and Eliza Hall Institute, AUSTRALIA

## Abstract

**Background:**

Primarily impacting poor, rural populations, the zoonotic malaria *Plasmodium knowlesi* is now the main cause of human malaria within Malaysian Borneo. While data is increasingly available on symptomatic cases, little is known about community-level patterns of exposure and infection. Understanding the true burden of disease and associated risk factors within endemic communities is critical for informing evidence-based control measures.

**Methodology/Principal findings:**

We conducted comprehensive surveys in three areas where *P*. *knowlesi* transmission is reported: Limbuak, Pulau Banggi and Matunggung, Kudat, Sabah, Malaysia and Bacungan, Palawan, the Philippines. Infection prevalence was low with parasites detected by PCR in only 0.2% (4/2503) of the population. *P*. *knowlesi* PkSERA3 ag1 antibody responses were detected in 7.1% (95% CI: 6.2–8.2%) of the population, compared with 16.1% (14.6–17.7%) and 12.6% (11.2–14.1%) for *P*. *falciparum* and *P*. *vivax*. Sero-prevalence was low in individuals <10 years old for *P*. *falciparum* and *P*. *vivax* consistent with decreased transmission of non-zoonotic malaria species. Results indicated marked heterogeneity in transmission intensity between sites and *P*. *knowlesi* exposure was associated with agricultural work (OR 1.63; 95% CI 1.07–2.48) and higher levels of forest cover (OR 2.40; 95% CI 1.29–4.46) and clearing (OR 2.14; 95% CI 1.35–3.40) around houses. Spatial patterns of *P*. *knowlesi* exposure differed from exposure to non-zoonotic malaria and *P*. *knowlesi* exposed individuals were younger on average than individuals exposed to non-zoonotic malaria.

**Conclusions/Significance:**

This is the first study to describe serological exposure to *P*. *knowlesi* and associated risk factors within endemic communities. Results indicate community–level patterns of infection and exposure differ markedly from demographics of reported cases, with higher levels of exposure among women and children. Further work is needed to understand these variations in risk across a wider population and spatial scale.

## Author summary

*Plasmodium knowlesi* is a species of malaria parasite found in wild macaque populations which is now the main cause of human malaria in Malaysian Borneo. Spread from macaques to people through infected mosquitoes, human *P*. *knowlesi* malaria cases have primarily been reported in adult men working in forests or plantations. However, little data is available on the extent of asymptomatic infections or people exposed to *P*. *knowlesi* not reporting to clinics. We conducted comprehensive surveys of three case study communities in Malaysian Borneo and Palawan, the Philippines with varying numbers of *P*. *knowlesi* cases reported. In addition to testing for infection, we measured species-specific antibody responses to *P*. *knowlesi* and other malaria species to identify exposed individuals. Few asymptomatic infections were detected and varying levels of *P*. *knowlesi* exposure was detected between sites. *P*. *knowlesi* exposure was identified in both men and women and associated with farm work and forest and clearing around the house. Spatial patterns and risk factors for *P*. *knowlesi* differed from other malaria species, highlighting the need for *knowlesi* specific disease control measures. Results suggest more people are exposed to *P*. *knowlesi* than are identified at clinics and exposure to *P*. *knowlesi* may occur in different demographic groups and geographic areas than previously reported.

## Introduction

After the initial recognition of a large number of human cases of the zoonotic malaria *Plasmodium knowlesi* in 2004 and advent of routine diagnosis of malaria cases by molecular methods, increasing numbers of human *P*. *knowlesi* cases have been reported in Southeast Asia and *P*. *knowlesi* is now the most common cause of human malaria in Malaysian Borneo [1–3]. Although regional control programmes have reduced the incidence of other malaria species in Malaysia and the Philippines, such as *P*. *falciparum* and *vivax*, the emergence of *P*. *knowlesi* presents a challenge to malaria elimination programmes. Despite increasing amounts of data available for symptomatic malaria cases presenting at hospital facilities, little is known about patterns of *P*. *knowlesi* exposure and infection at a community level [4].

Effectively targeting resources to identify and control *P*. *knowlesi* requires a detailed understanding of environmental and social risk factors. Carried by long and pig-tailed macaques (*Macaca fasicularus* and *M*. *nemestrina*), environmental changes affecting contact between people, mosquito vectors and simian hosts are believed to contribute to this apparent emergence of *P*. *knowlesi* in people [5, 6]. *Anopheles balabacensis*, the main knowlesi vector, has been associated with forest environments but is also found in peridomestic and agricultural areas [7, 8]. Associations between deforestation and increases in village-level incidence have been shown for clinical cases but this may not fully reflect exposure in the wider community [9]. Additionally, multiple studies have reported asymptomatic *P*. *knowlesi* infections, including in women and children, demographic groups comprising a minority of cases reported to facilities [10–14].

Patterns of community-level exposure can be assessed by the prevalence of specific antibodies against malaria parasites; these antibodies reflect exposure to previous infection and can be used to characterise the level of transmission and identify areas or groups with higher transmission [15]. These serological markers may be particularly useful in low transmission settings, where the probability of detecting infections is low [16]. Seroconversion rates derived from age specific sero-prevalence have also been shown to be closely correlated with more traditional measures of malaria transmission intensity, such as entomological inoculation rates or parasite prevalence, and can be used to identify differences in spatial patterns in transmission [17, 18]. Further, as these antibody responses represent exposure over time, longer term transmission patterns and temporal changes in transmission can be evaluated [19]. There are an increasing number of reagents for serological studies available for both *P*. *falciparum* and *P*. *vivax* e.g. [17, 20, 21]but antigens specific for *P*.*knowlesi* have only recently been described[22].

**Fig 1:**
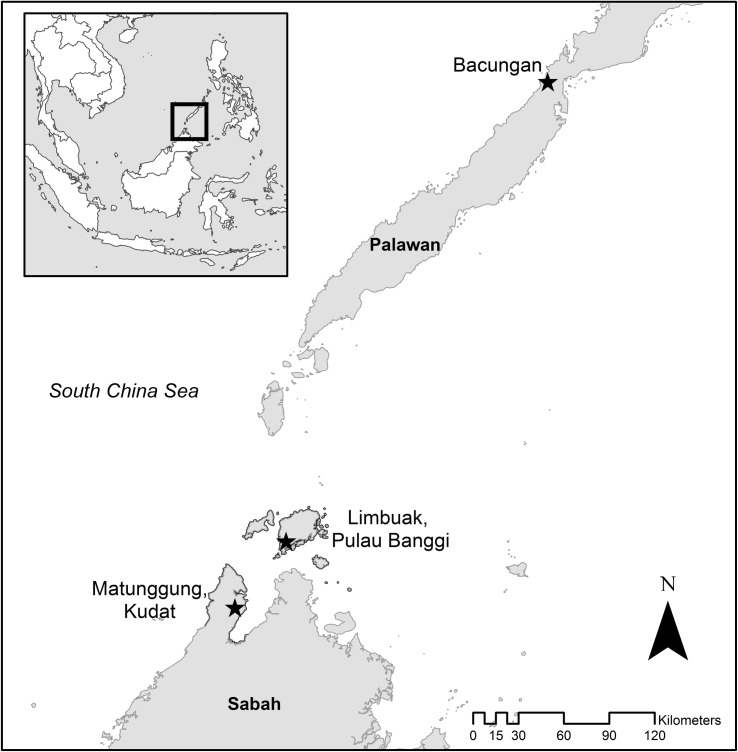
Study site locations in Matunggong, Kudat and Limbuak, Pulau Banggi in Sabah, Malaysia and Bacungan, Palawan, Philippines.

This study aimed to characterise these community level patterns of serological exposure to and prevalence of asymptomatic parasitemia of *P*. *knowlesi* and other malaria species in three case study communities where *P*. *knowlesi* transmission has been reported; a largely deforested and highly fragmented site at Matunggong, Kudat, an area with large patches of secondary forest bordering large scale clearing for an oil palm plantation in Limbuak, Pulau Banggi in Sabah, Malaysia and an area with intact secondary forest and some remaining primary forest in Bacungan, Palawan, The Philippines (Fig 1). These areas were selected as areas representative of locations were *P*. *knowlesi* transmission is occurring based on district hospital reports and were the sites of integrated entomology, primatology and social science studies within a wider research programme on risk factors for *P*. *knowlesi* (http://malaria.lshtm.ac.uk/MONKEYBAR). *P*. *knowlesi* is the main cause of reported human malaria in both the Matunggong and Limbuak sites while only few sporadic *P*. *knowlesi* cases have been reported from Bacungan [23–25]. Based on reporting of symptomatic cases to the national malaria programmes, the annual parasite incidence per 1000 people for *P*. *knowlesi* in 2014 was 12 for Matunggong, 2 for Limbuak and 0 for Bacungan.

## Methods

### Ethics approval and informed consent

This study was approved by the Medical Research Sub-Committee of the Malaysian Ministry of Health (NMRR-14-713-21117), the Institutional Review Board of the Research Institute for Tropical Medicine, Philippines and the Research Ethics Committee of the London School of Hygiene and Tropical Medicine (8340). Written informed consent was obtained from all participants or parents or guardians and assent obtained from children under 18 in this study and all methods were performed in accordance with relevant guidelines and regulations.

### Sampling methods

This study involved comprehensive sampling of all individuals residing within the study areas. Study sites were selected based on the locations of previously reported clinical *P*. *knowlesi* cases and all households within these communities were enumerated and geo-located. All individuals were asked to participate in the study and consenting individuals were interviewed on demographic characteristics, movement patterns, malaria prevention methods and land use practices. Individuals were excluded if they were less than 3 months old, had not primarily resided in the area for the past month or could not be reached after three attempts to contact them, including during evenings and weekends. Finger-prick blood samples were collected to test for malaria infection and exposure; these included blood smears to detect malaria parasites by microscopy and approximately 200μl whole-blood specimens collected in a tube containing EDTA (Becton-Dickinson, Franklin Lakes, New Jersey) and three 20μl spots stored on filter paper (3MM, Whatman, Maidstone, United Kingdom). Filter paper was dried and stored with desiccant at 4°C.

### Detection of malaria infection

All blood smears were examined by trained malaria microscopists. DNA was extracted from filter paper or 10 μl blood pellets using the Chelex-100 boiling method and a nested polymerase chain reaction (PCR) method targeting the *Plasmodium* small subunit ribosomal RNA (ssRNA) was used to identify malaria infected individuals, as described by [10, 26]. This assay used the genus-specific primers rPLU1 (5’-TCA AAG ATT AAG CCA TGC AAG TGA-3’) and rPLU5 (5’-CCT GTT GTT GCC TTA AAC TTC-3’) for nest 1 and rPLU3 (5’-TTT TTA TAA GGA TAA CTA CGG AAA AGC TGT-3’) and rPLU4 (5’-TAC CCG TCA TAG CCA TGT TAG GCC AAT ACC-3’) for nest 2. Thermal cycling conditions for primary and nested PCRs were 35 cycles at 94°C, 60°C and 72°C. Samples positive for the *Plasmodium* genus were then screened using species specific primers targeting the ssRNA region; for *P*. *knowlesi* these included PkF1140 (5’-GATTCATCTATTAAAAATTTGCTTC-3’) and PkR1150 (5' GAGTTCTAATCTCCGGAGAGAAAAGA 3') for 35 cycles at 50°C, 72°C and 94°C. All products were visualised on a 2% agarose gel. PCR for malaria infection was performed at laboratories at the Universiti Sabah Malaysia in Malaysia and Research Institute for Tropical Medicine in the Philippines, with PCR validation of a subset of samples at the London School of Hygiene and Tropical Medicine in the UK.

### Serological detection of exposure

Enzyme-linked immunosorbent assays (ELISA) were performed as previously described [27]. Briefly, 3 mm disc was excised from each dried blood spot and incubated in reconstitution buffer (PBS/tween with sodium azide) overnight at 4°C. Antibodies were eluted from the blood spots equivalent to a 1:100 dilution of whole blood or a 1:200 dilution of serum [16]. Antibody responses were measured against apical membrane antigen-1 or the 19 kDa fragment of merozoite surface protein-1 for *P*. *vivax* (PvAMA-1 and PvMSP-1_19_, respectively), *P*. *falciparum* (PfAMA-1 (PMID: 17192270; PMID: 19165323) and PfMSP-1_19_ (PMID: 8078519) and *P*.*knowlesi* SERA3 antigen 2[22]. The *Pk* serine repeat antigen (SERA) 3 antigen 2 (PKNH_0413400; chromosome 4) is a novel recombinant protein, N-terminally located between positions 826–998 aa, inclusive. SERA3 (1079 aa) belongs to a multigene family whose members encode a papain-like cysteine protease domain (ref: PMID: 21423628). In *P falciparum*, the N-terminal domain of SERA 5 is showing promise as a potential vaccine candidate (ref: PMID: 24886718, PMID: 27343834). The recombinant protein was expressed in *Escherichia coli* and affinity purified by a GST tag. Knowlesi -exposed hospital clinical case control samples showed antigen specific reactivity to the SERA3 antigen 2 recombinant when compared to responses from European malaria naïve and Ethiopian vivax-exposed serum samples (Herman et al. submitted) Eluates were tested in duplicate at a final concentration of 1:1000 for all antigens except 1:2000 for PfAMA-1. In addition, blank wells and a dilution series of the appropriate positive plasma pool were added per plate. Positive controls based on a hyper-immune endemic adult Tanzanian pool (PMID: 15792998), a lyophilised anti-malaria patient sample (NIBSC, UK; 72/96) and pooled *Pk*-exposed hospital serum samples were used to assay for *P*. *falciparum*, *P*. *vivax* and *P*. *knowlesi* antigens, respectively. Polyclonal rabbit anti-human IgG-HRP (Dako, Denmark) was used at 1/15,000 dilution and plates were developed using TMB (One component HRP microwell substrate, Tebu-bio). Optical density (OD) values were measured at 450 nm with a microplate reader. Values in excess of 1.5 CV between duplicates were considered fails and re-ran. OD values were corrected by subtracting the background of the blank well per plate. For *P*. *falciparum* and *P*. *vivax* OD readings, values were normalised between plates using a standardised control. Normalisation was not done for *P*. *knowlesi* results due to the lack of standard control. All serological analysis was performed at the Universiti Malaysia Sabah and the London School of Hygiene and Tropical Medicine.

### Environmental classification

**Fig 2:**
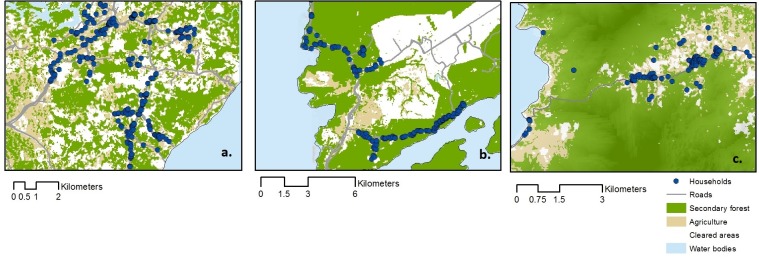
Land use classification of study sites. a. Highly deforested and fragmented site at Matunggong, Kudat, Sabah, Malaysia; b. Some forested area bordering large scale clearing in Limbuak, Pulau Banggi, Sabah, Malaysia; c. Mostly intact forest in Bacungan, Palawan, Philippines.

All households and roads within these areas were geo-located using a hand-held GPS (global positioning system). Land cover maps were derived from LANDSAT 8 30m resolution satellite images [28] and supervised classification was performed using random forests [29, 30]. In order to generate training data, high- resolution aerial images of areas within study sites were produced using the Sensefly eBee unmanned aerial vehicle flown at 400 metres above ground level (UAV or drone; Sensefly, Cheseaux-sur-Lausanne, Switzerland) and processed using Postflight Terra 3D (Pix4D SA, Lausanne, Switzerland) as described by [31]. These data were manually digitised and classified as forest, agricultural land (including cropland and agroforestry such as rubber and palm oil), open areas and water bodies. Additional data on elevation, aspect and slope was extracted from the ASTER global digital elevation model [32]. All data were resampled to 30m per pixel and datasets including topographic variables, distance from roads and houses, normalised differential vegetation indices (NDVI) and Landsat satellite data were included in the initial model. Random forest models were run using 10,000 trees to ensure stability and were run iteratively with least predictive variables excluded at every run [33]. A random subset of the training data for each site was withheld to independently validate the classification; estimated classification accuracy was 88%, 97% and 85% for Matunggung, Limbuak and Palawan respectively (Fig 2).

These classified land cover maps were used to calculate distance from the household to the forest edge. The proportions of different land types surrounding all households were evaluated for 100m, 500m and 1000m buffer radii. Additionally, the level of forest fragmentation was assessed within 500m and 1000m of each household; this was represented as the ratio of forest perimeter to forest area as described by [34]. All geographic data was stored and visualised in a Geographic Information System using ArcGIS (ArcGIS, Redlands, USA) and all other analysis was performed using R statistical software (R Foundation for Statistical Computing, Vienna, Austria, http://www.R-project.org).

### Statistical analysis and data management

Questionnaire data was collected electronically using Pendragon Forms VI (Pendragon Software Corporation, Chicago, USA) and analysed using R statistical software. To define sero-positive individuals, mixture models were fit for normalised optical densities (ODs), with the distribution of ODs modelled as two Gaussian distributions. Cut off values to define sero-prevalence for each antigen were defined as the mean OD of the sero-negative population plus 3 standard deviations for *P*. *falciparum* and *P*. *vivax* as described by [16]. For the *P*.*knowlesi* antigen a more parsimonious cutoff value was defined as the mean OD plus 5 standard deviations due to a lack of prior data. Because the assays were run in different laboratories, cut off values were defined separately for each antigen, malaria species and location (Palawan and Sabah). For *P*. *falciparum* and *P*. *vivax*, individuals were considered positive if they were positive for either MSP-1 and/or AMA-1. Reversible catalytic models were fit to age sero-prevalence data using maximum likelihood methods; these models were then used to generate age sero-prevalence curves and estimate the seroconversion rate (SCR) [17]. Evidence of historical changes in transmission was explored by using profile likelihood plots. Models with two SCR were assessed by likelihood ratio tests and used if the fit was significantly better (p < 0.05) than models with a constant seroconversion rate [19]. Models were fit separately for each parasite species and site.

Risk factors associated with *P*. *knowlesi* sero-positivity were evaluated using multivariate logistic regression, with household included as a random effect to account for correlation between individuals from the same household. An additional model was developed to compare individuals sero-positive for *P*. *knowlesi* with those sero-positive for non-zoonotic malaria species. Explanatory variables included age, gender, site, individual and household level farming activities, residence in the area, elevation and distance to forest. Additionally, the proportions and configuration of different land types were extracted for each household at 100m, 500m and 1000m radii and categorised as greater or less than 30% coverage within a specific radius in the final model. Univariate analysis was conducted for all explanatory variables and variables with p < 0.2 were included in multivariate analyses. For highly correlated variables (such as land cover proportions at different radii), single variables were selected based on marginal increases in Akaike Information Criterion (AIC). The final adjusted models were developed by retaining all variables significant at a 0.05 level and variables were added in a forward stepwise fashion to check for interactions. Potential residual spatial autocorrelation of exposure to *P*. *knowlesi* was assessed separately for all sites using Moran’s I.

Correlation between spatial patterns of exposure to *P*. *knowlesi* and nonzoonotic malaria species was explored through correlograms, plots of spatial autocorrelation with lag distances. First, ODs were log-transformed and adjusted for age by linear regression as described by [18]. For each site, cross-correlograms of antibody responses to *P*.*knowlesi* and each other antigen were plotted. Correlation ranges were determined by significance values (p < 0.05) of individual bins of lag distances of 500m. Pairwise correlation between antibody responses was determined using a simple Mantel test to test the significance of associations [35, 36].

## Results

The total populations resident in the sites were 1260 in Matunggong, 1009 in Limbuak and 686 in Bacungan. Surveys were conducted from October 2014 to January 2015 in Limbuak (n = 795) and Matunggong (n = 1162) sites in Sabah and in September 2014 in Bacungan, Palawan (n = 546). During this time, no clinical *P*. *knowlesi* cases were reported from the Bacungan study site while one *P*. *knowlesi* case was reported in the Limbuak site and two cases were reported in Matunggong site. The median age of participants was 24 years (age range 3 months– 99 years) and similar proportions of men and women were sampled in all study sites. While only 22% (538/2503) of individuals reported their primary occupation as farming or plantation work, the majority of individuals (74%; 1846/2503) reported their household engaged in some agricultural activities (Table 1). The proportion of forest cover within 1km of the houses in each site ranged from 39% in Matunggong, 55% in Bacungan to 82% in Limbuak (Fig 2). The Matunggong site was the most highly fragmented, with a forest perimeter to area ratio of 0.03 compared to 0.01 in Bacungan and 0.005 in Limbuak.

**Table 1:** Demographic and environmental characteristics of included participants.

		Limbuak, Pulau Banggi (n = 795)	Matunggong, Kudat (n = 1162)	Bacungan, Palawan (n = 546)
**Demographic factors**				
Gender				
	Female, % (n)	52.5 (417)	51.8 (602)	43.6 (238)
	Male, % (n)	47.5 (378)	48.2 (560)	56.4 (308)
Age in years, median (IQR)		22 (9–44)	25 (10–47)	25 (11–44)
Farming or plantation work as main occupation, % (n)		14.2 (113)	30.6 (356)	12.6 (69)
Household farm activities, % (n)		68.1 (542)	88.6 (1030)	50.1 (274)
Stay overnight outside village, % (n)		8.2 (65)	13.6 (161)	29.5 (161)
**Environmental factors**				
Elevation (metres above sea level), median (IQR)		11 (8–15)	50 (35–75)	84 (77–114)
Distance to forest edge (metres), median (IQR)		30 (30–60)	95 (68–120)	67 (30–108)
Proportion of cleared areas around house (%), median (IQR)				
	Within 100m	0.43 (0.21–0.65)	0.63 (0.46–0.74)	0.39 (0.26–0.61)
	Within 500m	0.14 (0.10–0.24)	0.38 (0.28–0.47)	0.22 (0.16–0.26)
	Within 1000m	0.14 (0.09–0.17)	0.37 (0.31–0.39)	0.18 (0.16–0.20)
Proportion of agriculture around house (%), median (IQR)				
	Within 100m	0.14 (0.03–0.31)	0.33 (0.23–0.49)	0.43 (0.32–0.60)
	Within 500m	0.06 (0.05–0.14)	0.38 (0.28–0.48)	0.39 (0.36–0.42)
	Within 1000m	0.05 (0.03–0.10)	0.31 (0.24–0.36)	0.37 (0.29–0.39)
Proportion of forest around house (%), median (IQR)				
	Within 100m	0.31 (0.12–0.50)	0.03 (0–0.08)	0.08 (0–0.20)
	Within 500m	0.71 (0.59–0.81)	0.22 (0.13–0.34)	0.37 (0.32–0.43)
	Within 1000m	0.79 (0.76–0.86)	0.33 (0.27–0.39)	0.44 (0.40–0.52)
Forest area to perimeter ratio around house, median (IQR)				
	Within 500m	0.02 (0.01–0.02)	0.05 (0.04–0.06)	0.04 (0.03–0.04)
	Within 1000m	0.01 (0.01–0.01)	0.03 (0.03–0.04)	0.04 (0.03–0.04)

### Infection with malaria

Two microscopy positive individuals were identified from the Matunggong, Kudat site; these were both subsequently identified as *P*. *knowlesi* mono-infections by PCR. All PCR infections were re-confirmed at the laboratory in the U.K. Both of these individuals were male plantation workers (ages 21 and 25) residing in the same household. An additional two individuals in Matunggong were microscopy negative but identified as *P*. *knowlesi* infected by molecular methods; these included a three year old girl and 33 year old woman residing in different villages within the study site. Only one out of these four infected individuals identified self-reported history of fever. None of the survey participants in either the Limbuak or Bacungan sites were positive by microscopy or PCR and no infections with any other malaria species were identified in any participants.

### Serological assessment of exposure to *P*. *knowlesi*

**Fig 3:**
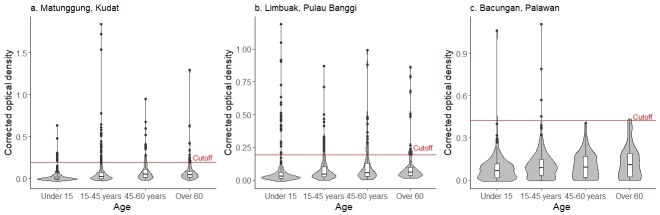
Violin plots of antibody density by age group: a. Matunggong, Kudat, b. Limbuak, Pulau Banggi, c. Bacungan Palawan.

Overall, 7.1% (178/2503) of the population surveyed was seropositive to *P*. *knowlesi* (Fig 3). Exposure varied substantially between study sites, with the highest *P*. *knowlesi* antibody prevalence detected in Limbuak, Pulau Banggi (11.7%; 93/795) followed by 6.8% (79/1162) in Matunggong Kudat. Bacungan, Palawan had the lowest sero-prevalence (1.1%; 6/546). Similar reactivity to *P*. *knowlesi* was observed in men (optical density (OD): med: 0.035, IQR: 0.006–0.094) and women (OD: median: 0.035, IQR: 0.007–0.089) and gender was not significantly associated with *P*. *knowlesi* sero-positivity (OR: 0.99, 95% CI: 0.71–1.37, p = 0.95).

Antibody prevalences to *P*. *falciparum* and *P*. *vivax* were higher in all sites, with 16.1% (364/2266) of individuals sero-positive to one or both *P*. *falciparum* antigens and 12.6% (270/2141) positive for one or more *P*. *vivax* antigens. Sero-prevalence to *P*. *falciparum* was 16.9% (196/1162) in Matunggong, 13.5% (107/795) in Limbuak and 10.4% (61/587) in Bacungan. In contrast, reactivity to *P*. *vivax* was highest in Limbuak (16.7%; 133/795) with sero-prevalences of 6.9% (80/1162) and 9.7% (57/587) in Matunggong and Bacungan respectively. Due to insufficient samples and non-systematic errors in labelling, results for all antigens were not available for all individuals. Out of individuals with complete test results for all antigens, 25.7% (499/1941) of participants were sero-positive to at least one species of malaria and 7.9% (54/1941) were sero-positive for two or more malaria species. Of individuals exposed to *P*. *knowlesi*, 29.7% (53/ 178) were also positive for *P*. *falciparum* or *P*. *vivax* antigens. There was no evidence of correlation between *P*. *knowlesi* and other antigens tested (S1 Fig).

**Fig 4:**
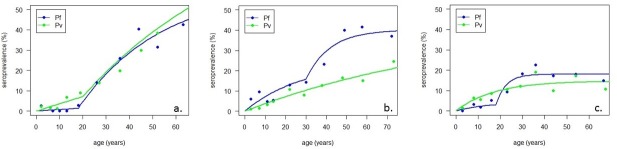
Seroprevalence curves for each location used to calculate SCRS (λ). a. Pulau Banggi, Sabah (Pf λ1: 0.0196 (0.0078–0.0492); Pf λ2: 0.0008 (0.0002–0.0031); Pv λ1: 0.0185 (0.0136–0.0248)) b. Matunggong, Kudat ((Pf λ1: 0.0588 (0.0198–0.1746); Pf λ2: 0.0085 (0.0063–0.0116); Pv λ: 0.0039 (0.0024–0.0064)) c. Bacungan, Palawan (Pf λ1: 0.1441 (0.0175–1.1892); Pf λ2: 0.0031 (0.0012–0.0086); Pv λ: 0.0086 (0.0044–0.0166)).

Sero-prevalence was positively associated with increases in age for all antigens tested. However, despite this, seroreactivity, including individuals with high antibody titres (S2 Fig), was still detected in the youngest age groups and 4.2% (39/921) individuals under 15 years had antibodies to *P*. *knowlesi*, 3.5% (29/821) had antibodies to *P*. *falciparum* and 2.9% (23/792) to *P*. *vivax*. Changes in age sero-prevalence were more pronounced for *P*. *falciparum* and *P*. *vivax*, with 32.9% (78/237) and 28.1% (64/228) reactivity to *P*. *falciparum* and *P*. *vivax* in individuals over the age of 60 years. In contrast, antibodies for *P*. *knowlesi* were detected in 9.4% (25/265) of individuals over 60 years old and the highest sero-prevalence was detected in adults from 45–60 years old (11.6%; 43/370). As reactivity to *P*. *knowlesi* was low and not evenly distributed through the population, seroconversion rates (SCR) for *P*. *knowlesi* could not be calculated. Historical changes in falciparum transmission intensity were apparent in all sites and SCR models fitted with two forces of infection suggest substantial reductions in *P*. *falciparum* transmission occurred 18–30 years ago (p < 0.05) (Fig 4). Strong evidence of decreased transmission intensity for *P*. *vivax* was only seen in Limbuak, where transmission decreased over 25-fold in the past 20 years.

### Factors associated with *P*. *knowlesi* sero-positivity

Demographic and environmental characteristics of survey participants are summarised in (Table 1). In addition to age and site, reporting farm or plantation work as a primary occupation was positively associated with *P*. *knowlesi* sero-positivity (Table 2). Higher proportions of forest cover within 1km of the household and cleared areas within 500m of the house were both associated with increased odds of *P*. *knowlesi* positivity. While forest fragmentation, elevation and agricultural land around the house were significant within the univariate analysis, none of these variables were significant in the final multivariate model (Supplementary information, S1 Table). Similar proportions of men and women reacted to *P*. *knowlesi* in all sites and gender was not associated with sero-positivity.

**Table 2:** Multivariate analysis of risk factors for *P*. *knowlesi* seropositivity. (comparison of *P*. *knowlesi* exposed individuals with non-exposed individuals).

		Adjusted OR (95% CI)	P value
Age			
	Under 15 years	-	< 0.001
	15–45 years	2.05 (1.30–3.22)	
	45–60 years	2.94 (1.70–5.11)	
	Over 60 years	2.46 (1.32–4.58)	
Site			
	Palawan	-	< 0.001
	Mainland Kudat	4.30 (1.66, 11.15)	
	Pulau Banggi	10.83 (4.50, 26.10)	
Main occupation farm or plantation work			
	No	-	0.025
	Yes	1.63 (1.07, 2.48)	
Forest cover within 1km			0.004
	Less than 30%	-	
	Over 30% forest cover	2.40 (1.29, 4.46)	
Proportion of cleared/ open area within 500m of house			0.001
	Less than 30%	-	
	Over 30% cleared	2.14 (1.35, 3.40)	

Individuals reacting to *P*. *knowlesi* were more likely to be younger than individuals sero-positive for only non-zoonotic malaria species (Table 3). Forest cover was not associated with exposure to non-zoonotic malaria and malaria positive individuals residing in areas with high forest cover around the house had 4.86 (95% CI: 2.30–11.37) the odds of being positive for *P*. *knowlesi*. Similarly, cleared areas around the house were also positively associated with *P*. *knowlesi* cases compared to other malaria species.

**Table 3:** Multivariate analysis of risk factors for *P*. *knowlesi* seropositivity in malaria exposed individuals. (comparison of *P*. *knowlesi* exposed individuals with individuals exposed to other non-zoonotic malaria species).

		Adjusted OR (95% CI)	P value
Age			
	Under 15 years	-	0.05
	15–45 years	0.72 (0.37–1.39)	
	45–60 years	0.53 (0.26–1.06)	
	Over 60 years	0.38 (0.18–0.82)	
Site			
	Palawan	-	< 0.001
	Mainland Kudat	3.79 (1.50, 11.00)	
	Pulau Banggi	6.55 (2.88, 17.68)	
Proportion of forest within 1km of house			
	Less than 30%	-	< 0.001
	Over 30% cleared	4.86 (2.30, 11.37)	
Proportion of cleared/ open area within 500m of house			
	Less than 30%	-	0.001
	Over 30% cleared	2.70 (1.60, 4.66)	

Based on Moran’s I, there was no evidence of residual spatial autocorrelation for *P*. *knowlesi* antibody responses (Moran’s I p > 0.2 for all sites). There was no significant spatial correlation detected between age-adjusted antibody responses for *P*. *knowlesi* and other malaria species for either Matunggong or Limbuak (p > 0.30 for all pairwise comparisons). Comparisons between *P*. *knowlesi* and other malaria species could not be evaluated for Bacungan due to the low prevalence of *P*. *knowlesi* sero-positivity.

## Discussion

This is the first study to describe exposure to *P*. *knowlesi* through antigen specific antibody responses and associated risk factors and is one of few studies to assess *P*. *knowlesi* carriage prevalence at a community level. Results indicate spatial and temporal patterns of *P*. *knowlesi* transmission differ markedly from other non-zoonotic malaria species within the region. Although *P*. *knowlesi* sero-positivity was associated with some landscape attributes within these communities, extensive cross sectional surveys are needed to identify ecological risk factors across a broader geographic scale.

Sero-prevalence data indicate distinct heterogeneities in *P*. *knowlesi* transmission intensity between sites. Although formal comparisons between *P*. *knowlesi* infection and exposure could not be undertaken due to the low prevalence of parasite carriage, these geographical differences in transmission mirror hospital-based reporting rates in the study sites at Kudat, Pulau Banggi and Palawan [23–25]. These results also highlight the utility of serological techniques to identify differences in transmission intensity in settings where the sensitivity of parasite prevalence surveys is limited by the scarcity of infected individuals and suboptimal diagnostics. This is the first time these knowlesi-specific antigens have been used at a population level to assess species-specific exposure to malaria. Although high levels of homology between *P*. *knowlesi* and *P*. *vivax* indicate the possibility of cross reactivity between antigens, relatively low numbers of individuals were identified as sero-positive for both knowlesi and vivax (2%; 43/2102 individuals with results for both assays) and individuals could have been plausibly exposed to both species due to the co-endemicity of these species within this region. Additional work has been done to characterise response to *P*. *knowlesi* in vivax-exposed individuals and validate these antigens for population-based studies[22].

Changes in seroconversion rates can also reflect temporal changes in malaria transmission. In Sabah, state-wide malaria notification records describe dramatic decreases in clinical *P*. *falciparum* and *P*. *vivax* cases within the past 20 years following the scale up of malaria control and elimination programmes [2]. The Philippines has also reported a substantial decline in the number of malaria cases reported in the past few decades, most notably for *P*. *falciparum*[37]. These changes are evident in seroconversion rates to non-zoonotic malaria species from the 3 sites with over 5-fold difference between current and previous SCRs. *P*. *knowlesi* exposure was identified in children under 5 in all sites, suggesting recent or on-going transmission albeit at a low level. Further work is needed to refine *P*. *knowlesi* serological analysis to allow for antigenic variation, identify further antigenic targets and assess the differential responsiveness of individuals and longevity of antibody responses [38].

Despite these similarities between existing case data and community-level exposure to *P*. *knowlesi*, levels of exposure between different demographic groups varied markedly from clinical case reports. While clinical *P*. *knowlesi* has been commonly reported in adult men, men and women had similar antibody reactivity to *P*. *knowlesi* antigens in all sites [23, 39]. Within Kudat district, wide age distributions and family clusters of knowlesi cases have previously been described; however, from 2012–2015, 73% (84/115) and 77% (27/35) of all clinical cases reported from Kudat and Pulau Banggi respectively were men [23]. Asymptomatic knowlesi carriage has been detected in higher proportions of women by this study and other studies; however these results are extremely limited by sampling design and the small numbers of infected individuals detected [10, 12]. As forest and agricultural activities have been identified as risk factors for clinical *P*. *knowlesi* infection, more men could develop clinical infections due to higher exposure or number of bites; however, this requires further research to assess [40]. Larger scale population-based cross sectional surveys are needed to determine if these patterns occur in the wider community and if *P*. *knowlesi* affects groups which may be underrepresented by current passive surveillance systems.

*P*. *knowlesi* exposure was also associated with landscape factors. Both the proportion of forest cover and cleared areas around the household were positively associated with knowlesi sero-positivity, potentially reflecting the higher vectorial capacity and sporozoite rates reported in secondary forest within these study sites [7]. Although plantation or farm work as a primary occupation was associated with increased exposure and previous reports have described associations between *P*. *knowlesi* and forest activities, data on movement into different environments was not available for all survey participants [39, 41]. Instead, to explore the potential range of spatial interactions between people and mosquito vectors, proportions of habitat were evaluated at different buffer distances around houses. The significance of both clearing and forest areas at different radii suggests the importance of edge effects, transition areas between habitats where increased overlap of human, macaque and mosquito populations may occur [9, 42, 43]. Despite this, no associations were identified between metrics of fragmentation or distance to forest edges; this may reflect the limited environmental variation within these small spatial scales. Future studies could assess the importance of these variables across different ecotypes as well as collect more detailed data on the human movement into different environments, particularly during peak mosquito biting times.

These spatial patterns differed markedly from exposure to other non-zoonotic malaria species. Individuals with antibodies to *P*. *knowlesi* were more likely to reside in areas with higher proportions of forest cover; this may reflect differences in disease dynamics between species or temporal changes in transmission. Because of the longevity of antibody responses and the rapid rates of land use change within these areas, seroreactivity to non-zoonotic species is probably more likely to be associated with past rather than current environmental factors. The main mosquito vector species of *P*. *knowlesi*, *Anopheles balabacensis*, was historically incriminated as the main vector of other human malaria species within these same areas [44, 45]. While these vectors have been primarily associated with forest habitats, high vector densities have also been reported in small scale farms and other habitat types [7, 42, 46]. Deforestation and increased application of vector control measures may have triggered changes in vector composition and biting preferences; similarly, habitat changes and encroachment of human settlements into forest areas may have also led to changes in macaque population densities and closer contact between macaques, people and mosquito vectors [6, 47].

The main limitations of this study are the non-randomised population sampling approach and limited geographical scale. While this study describes fine scale patterns of malaria exposure and infection within these three case study communities, these results cannot be generalised to extrapolate *P*. *knowlesi* risks across wider populations. As this study surveyed three relatively homogenous populations, there was minimal variation in environment, ethnicity, socioeconomic status and access to healthcare within each site. Identifying environmental and population-level risk factors will require randomised sampling across a wider ecological gradient; community level data on presence and absence of exposure and infection are required to understand spatial heterogeneity of disease transmission and develop and refine predictions of disease risk [48]. Additionally, extensive surveys of parasite prevalence may allow the application of genetic approaches to track parasite diversity and transmission and explore the roles of host and parasite genetic factors.

Despite these limitations, this study describes *P*. *knowlesi* infection and exposure within these communities and illustrates how serologic markers can be used to describe differences in transmission intensity between malaria species in low transmission settings. Results from these surveys indicate patterns of *P*. *knowlesi* exposure and infection within the community may be substantially different from cases detected by passive surveillance systems. Cross sectional surveys across a broader geographical scale are needed to describe spatial variation in transmission intensity and identify associated environmental and population-based risk factors. Integration of serology into these surveys would provide vital information on rare infections for control programmes [49].

## Supporting information

S1 Checklist STROBE checklist for observational studies. (DOCX)

S1 Fig Comparison of normalised optical densities for *P*. *knowlesi* and other antigens: Optical densities and cut offs for Sabah, Malaysia. (TIF)

S2 Fig Comparison of normalised optical densities for *P*. *knowlesi* and other antigens: Optical densities and cut offs for Palawan, Philippines. (TIF)

S3 Fig Violin plots of antibody density for *P*. *falciparum* by age group and site. a. *P*. *falciparum* AMA-1 antibody density; b. *P*. *falciparum* MSP-1 antibody density.
(TIF)

S4 Fig Violin plots of antibody density for *P*. *vivax* by age group and site. a. *P*. *vivax* AMA-1 antibody density; b. *P*. *vivax* MSP-1 antibody density.
(TIF)

S1 Table Proportions and univariate analysis of risk factors for *P*. *knowlesi* seropositivity. (DOCX)
