# Supplementary material for: Exposure and infection to Plasmodium knowlesi in case study communities in Northern Sabah, Malaysia and Palawan, The Philippines
Source: PLoS Negl Trop Dis. 2018 Jun 14;12(6):e0006432. doi: 10.1371/journal.pntd.0006432 (PMC6001952; doi:10.1371/journal.pntd.0006432)
Supplement: S1 Checklist — (DOCX) [file pntd.0006432.s001.docx]

STROBE Statement—checklist of items that should be included in reports of observational studies

|  | Item No. | Recommendation | Page  No. | Relevant text from manuscript |
| --- | --- | --- | --- | --- |
| **Title and abstract** | 1 | (*a*) Indicate the study’s design with a commonly used term in the title or the abstract | 2 | “comprehensive surveys in three areas” |
|  |  | (*b*) Provide in the abstract an informative and balanced summary of what was done and what was found | 2-3 | Abstract section |
| Introduction | | | |  |
| Background/rationale | 2 | Explain the scientific background and rationale for the investigation being reported | 4-5 | Introduction on *P. knowlesi* and lack of community wide exposure data |
| Objectives | 3 | State specific objectives, including any prespecified hypotheses | 5 | Lines 110 - 113 |
| Methods | | | |  |
| Study design | 4 | Present key elements of study design early in the paper | 6 | Lines 136 – 143 |
| Setting | 5 | Describe the setting, locations, and relevant dates, including periods of recruitment, exposure, follow-up, and data collection | 5, 10 | Lines 112 – 123, Lines 251 - 254 |
| Participants | 6 | (*a*) *Cohort study*—Give the eligibility criteria, and the sources and methods of selection of participants. Describe methods of follow-up  *Case-control study*—Give the eligibility criteria, and the sources and methods of case ascertainment and control selection. Give the rationale for the choice of cases and controls  *Cross-sectional study*—Give the eligibility criteria, and the sources and methods of selection of participants | 6 | Lines 140 - 144 |
|  |  | (*b*) *Cohort study*—For matched studies, give matching criteria and number of exposed and unexposed  *Case-control study*—For matched studies, give matching criteria and the number of controls per case |  |  |
| Variables | 7 | Clearly define all outcomes, exposures, predictors, potential confounders, and effect modifiers. Give diagnostic criteria, if applicable | 6- 9, 11 | Questionnaire data: Lines 140 – 143, Table 1; Environmental data: Lines 179 – 206, Outcome data: Lines 150 – 177, 210 - 219 |
| Data sources/ measurement | 8* | For each variable of interest, give sources of data and details of methods of assessment (measurement). Describe comparability of assessment methods if there is more than one group | 6 - 10 | Data sources Lines: 138 – 206, Methods of assessment: Lines 210 – 247 |
| Bias | 9 | Describe any efforts to address potential sources of bias | 10 | Development of multivariate model accounting for cofounders and assessment of collinearity: Lines 227 - 240 |
| Study size | 10 | Explain how the study size was arrived at | 6 | Comprehensive sampling (no study size calculated as included all individuals) |

Continued on next page

| Quantitative variables | 11 | Explain how quantitative variables were handled in the analyses. If applicable, describe which groupings were chosen and why | 10-11, 13-14 | Lines 230 – 240, Table 1-3 |
| --- | --- | --- | --- | --- |
| Statistical methods | 12 | (*a*) Describe all statistical methods, including those used to control for confounding | 10 | Lines 234 - 247 |
|  |  | (*b*) Describe any methods used to examine subgroups and interactions | 10 | Lines 236-239 |
|  |  | (*c*) Explain how missing data were addressed | NA | No missing data |
|  |  | (*d*) *Cohort study*—If applicable, explain how loss to follow-up was addressed  *Case-control study*—If applicable, explain how matching of cases and controls was addressed  *Cross-sectional study*—If applicable, describe analytical methods taking account of sampling strategy | NA | Sampling strategy comprehensive |
|  |  | (*e*) Describe any sensitivity analyses | NA |  |
| Results | | | | |
| Participants | 13* | (a) Report numbers of individuals at each stage of study—eg numbers potentially eligible, examined for eligibility, confirmed eligible, included in the study, completing follow-up, and analysed | 10-12 | Lines 250-253, 287 - 298 |
|  |  | (b) Give reasons for non-participation at each stage | 12 | Lines 292-294 |
|  |  | (c) Consider use of a flow diagram |  |  |
| Descriptive data | 14* | (a) Give characteristics of study participants (eg demographic, clinical, social) and information on exposures and potential confounders | 10 -11 | Lines 250 – 260, Table 1 |
|  |  | (b) Indicate number of participants with missing data for each variable of interest | NA | No missing data |
|  |  | (c) *Cohort study*—Summarise follow-up time (eg, average and total amount) | NA |  |
| Outcome data | 15* | *Cohort study*—Report numbers of outcome events or summary measures over time |  |  |
|  |  | *Case-control study—*Report numbers in each exposure category, or summary measures of exposure |  |  |
|  |  | *Cross-sectional study—*Report numbers of outcome events or summary measures | 11-12 | Lines 268, 279 – 297 |
| Main results | 16 | (*a*) Give unadjusted estimates and, if applicable, confounder-adjusted estimates and their precision (eg, 95% confidence interval). Make clear which confounders were adjusted for and why they were included | SI | Supplementary information |
|  |  | (*b*) Report category boundaries when continuous variables were categorized | 11, 13-14, SI | Tables |
|  |  | (*c*) If relevant, consider translating estimates of relative risk into absolute risk for a meaningful time period |  |  |

Continued on next page

| Other analyses | 17 | Report other analyses done—eg analyses of subgroups and interactions, and sensitivity analyses | 13, 14 | Seroconversion analysis: Lines 310-314, Fig 3; Spatial analysis: Lines 368 - 373 |
| --- | --- | --- | --- | --- |
| Discussion | | | | |
| Key results | 18 | Summarise key results with reference to study objectives | 16-18 | Lines 384 – 453 |
| Limitations | 19 | Discuss limitations of the study, taking into account sources of potential bias or imprecision. Discuss both direction and magnitude of any potential bias | 18-19 | Lines 455 – 466 |
| Interpretation | 20 | Give a cautious overall interpretation of results considering objectives, limitations, multiplicity of analyses, results from similar studies, and other relevant evidence | 19 | Lines 468 – 475 |
| Generalisability | 21 | Discuss the generalisability (external validity) of the study results | 19 | Lines 470 – 475 |
| Other information | |  | | |
| Funding | 22 | Give the source of funding and the role of the funders for the present study and, if applicable, for the original study on which the present article is based | Funding information |  |

*Give information separately for cases and controls in case-control studies and, if applicable, for exposed and unexposed groups in cohort and cross-sectional studies.

**Note:** An Explanation and Elaboration article discusses each checklist item and gives methodological background and published examples of transparent reporting. The STROBE checklist is best used in conjunction with this article (freely available on the Web sites of PLoS Medicine at http://www.plosmedicine.org/, Annals of Internal Medicine at http://www.annals.org/, and Epidemiology at http://www.epidem.com/). Information on the STROBE Initiative is available at www.strobe-statement.org.
