# Supplementary figures and images for: Exposure and infection to Plasmodium knowlesi in case study communities in Northern Sabah, Malaysia and Palawan, The Philippines
Source: PLoS Negl Trop Dis. 2018 Jun 14;12(6):e0006432. doi: 10.1371/journal.pntd.0006432 (PMC6001952; doi:10.1371/journal.pntd.0006432)

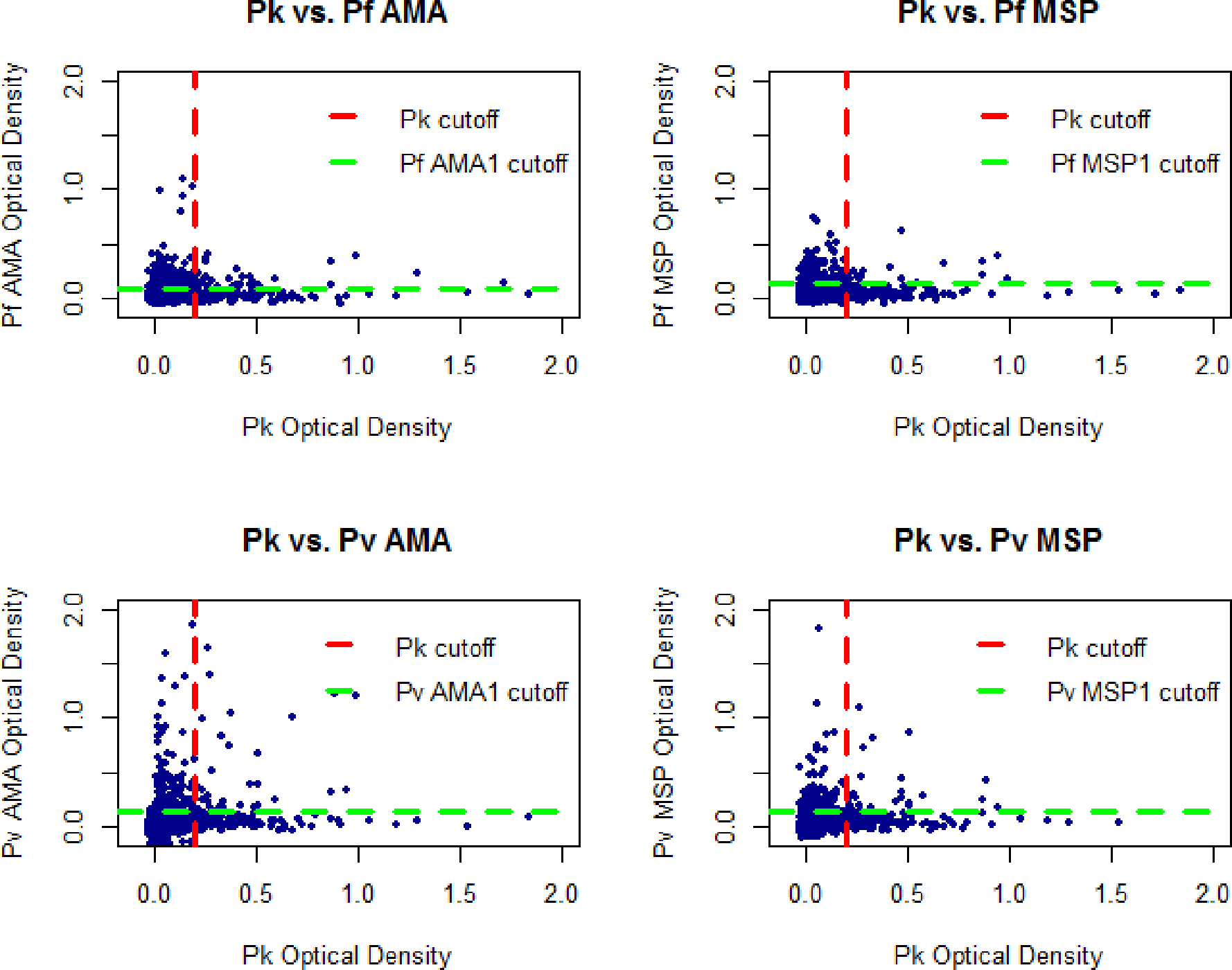

Supplement: S1 Fig — (TIF) [file pntd.0006432.s002.tif]

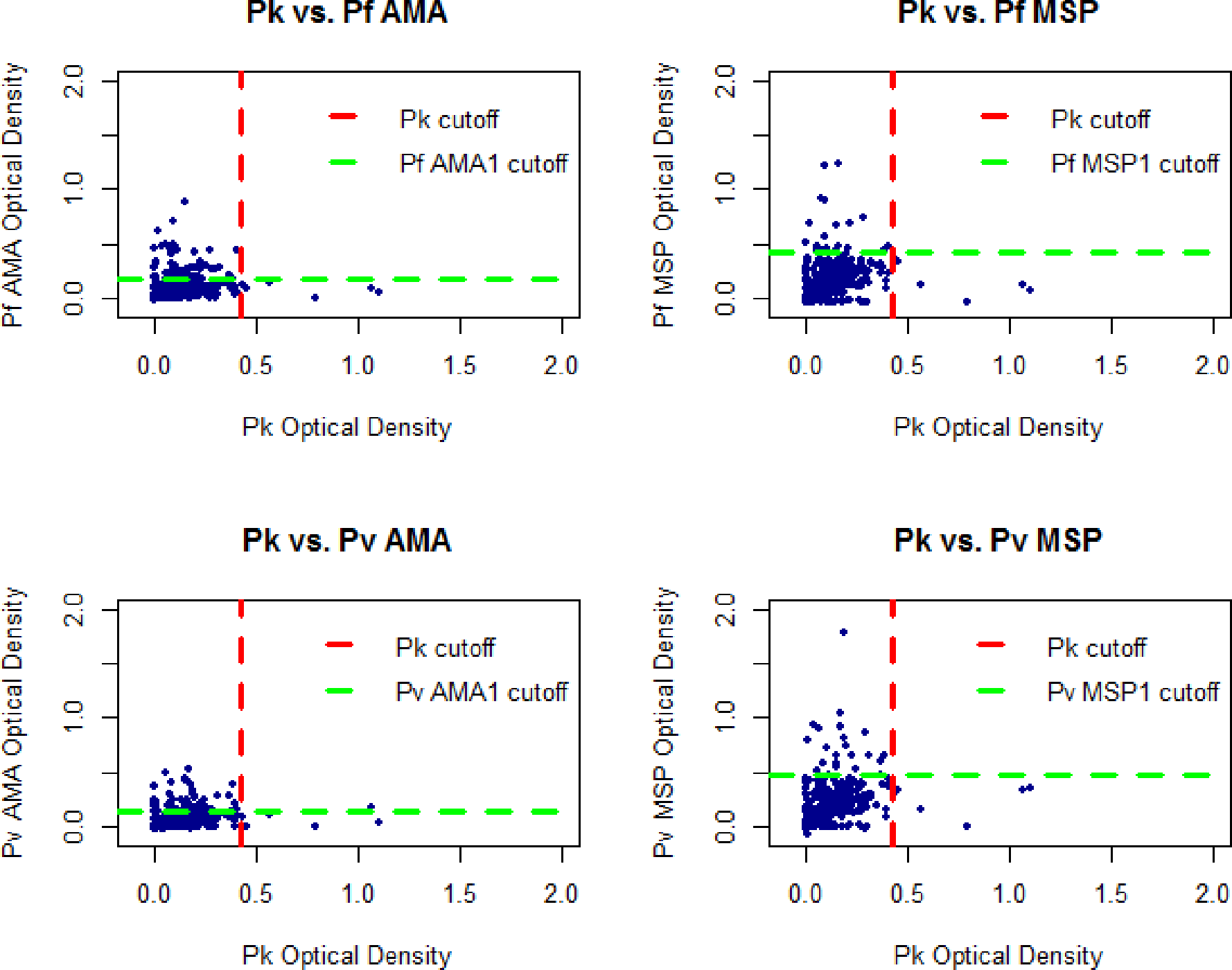

Supplement: S2 Fig — (TIF) [file pntd.0006432.s003.tif]

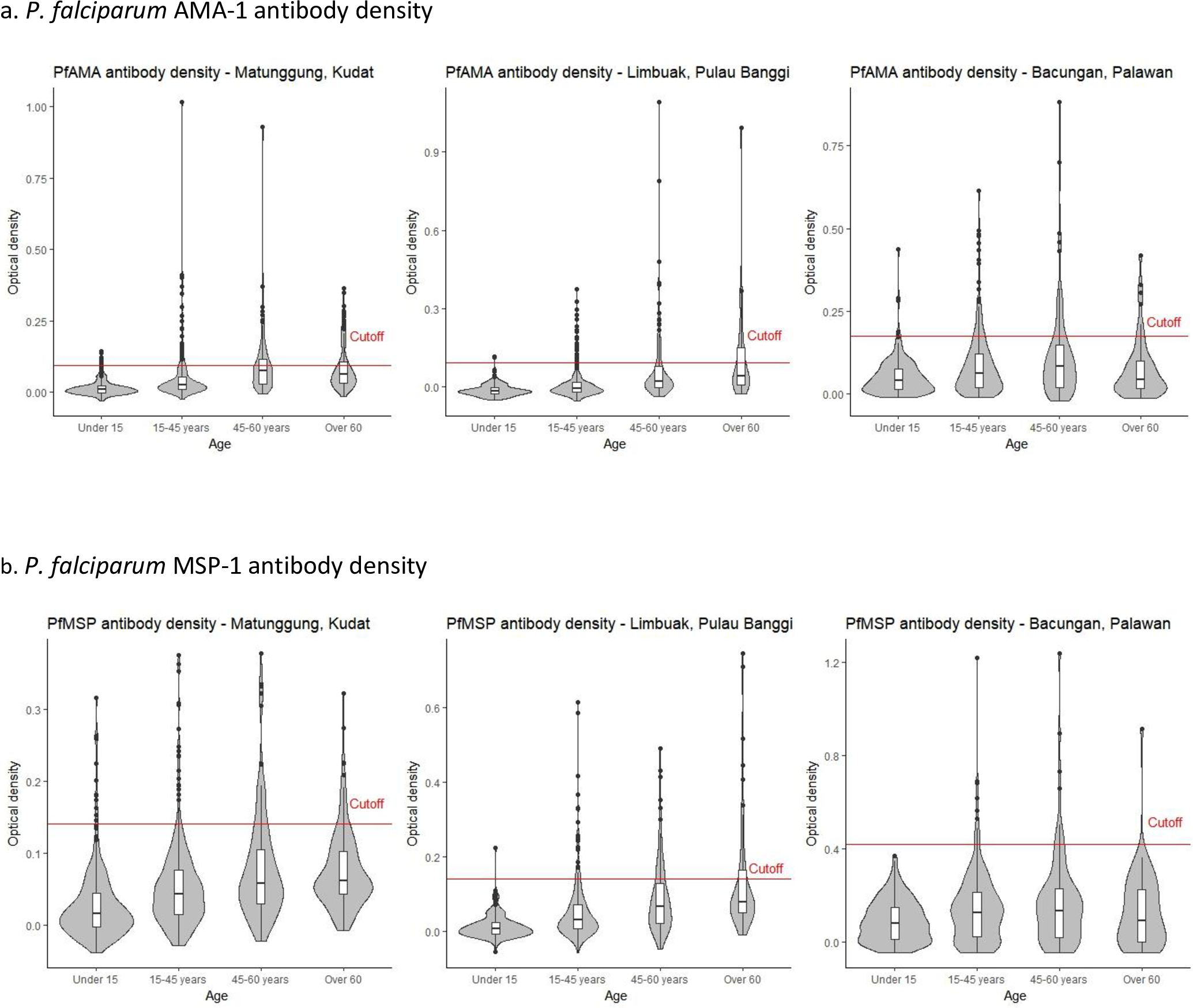

Supplement: S3 Fig — a. P. falciparum AMA-1 antibody density; b. P. falciparum MSP-1 antibody density. (TIF) [file pntd.0006432.s004.tif]

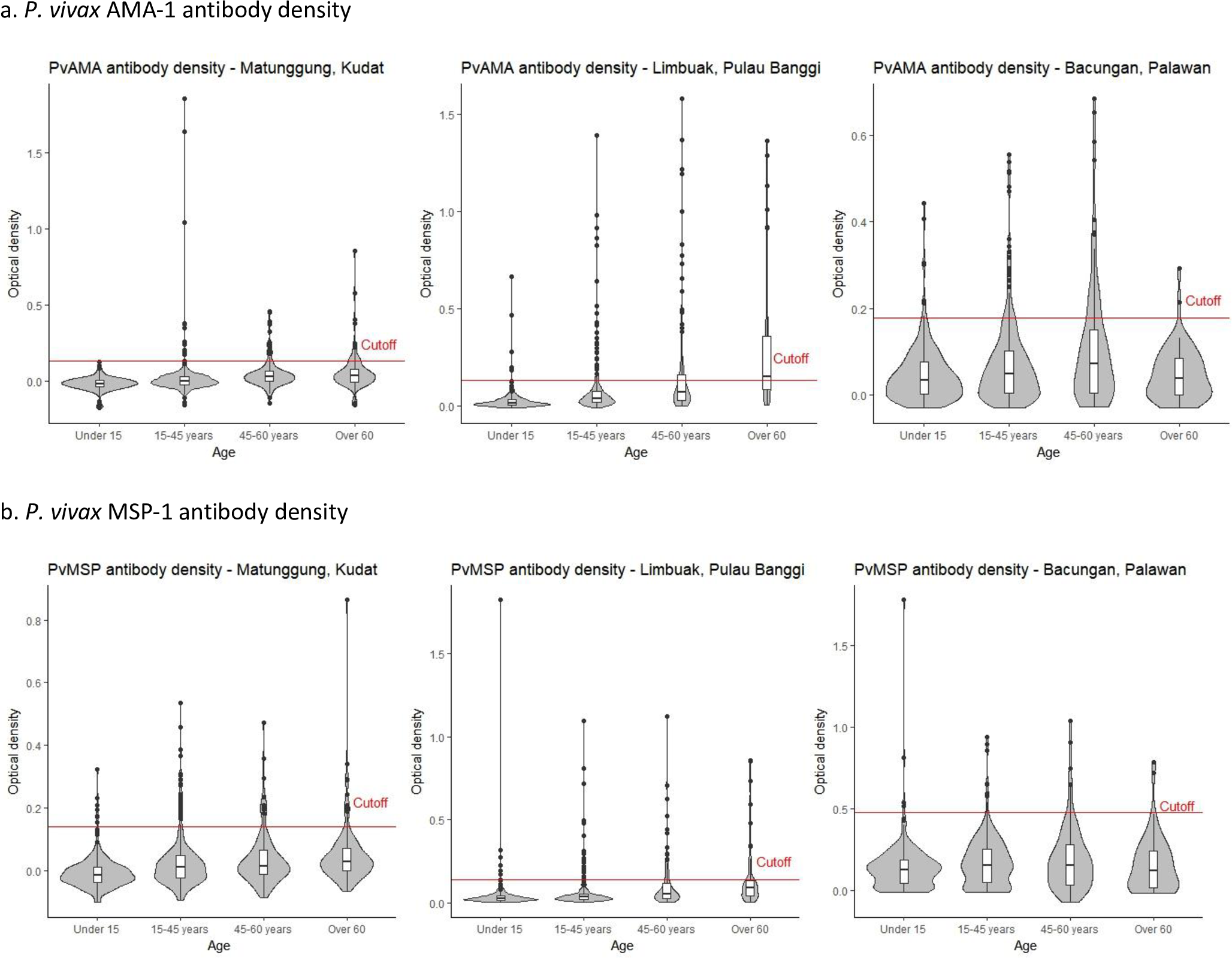

Supplement: S4 Fig — a. P. vivax AMA-1 antibody density; b. P. vivax MSP-1 antibody density. (TIF) [file pntd.0006432.s005.tif]
