# Supplementary material for: Exposure and infection to Plasmodium knowlesi in case study communities in Northern Sabah, Malaysia and Palawan, The Philippines
Source: PLoS Negl Trop Dis. 2018 Jun 14;12(6):e0006432. doi: 10.1371/journal.pntd.0006432 (PMC6001952; doi:10.1371/journal.pntd.0006432)
Supplement: S1 Table — (DOCX) [file pntd.0006432.s006.docx]

Table S1: Proportions and univariate analysis of risk factors for *P. knowlesi* seropositivity

|  | | **All** | | **Pk Seropositive** | | **Unadjusted OR (95%CI)*** | |
| --- | --- | --- | --- | --- | --- | --- | --- |
|  |  | N | % | N | % |  | P value |
| Total samples | | 2503 |  | 178 | 7.1% |  |  |
| Site | |  |  |  |  |  |  |
|  | Palawan | 546 | 21.8% | 6 | 1.1% |  | < 0.001 |
|  | Mainland Kudat  Banggi | 1162  795 | 46.4%  31.8% | 79  93 | 6.8%  11.7% | 7.02 (2.93, 16.86)  12.80 (5.31, 30.89) |  |
| Gender | |  |  |  |  |  |  |
|  | Female | 1257 | 50.2% | 90 | 7.2% |  | 0.95 |
|  | Male | 1246 | 49.8% | 88 | 7.1% | 0.99 (0.71, 1.37) |  |
| Age | |  |  |  |  |  |  |
|  | Under 15 years | 921 | 36.8% | 39 | 4.2% |  | < 0.001 |
|  | 15 – 45 years | 947 | 37.8% | 71 | 7.5% | 2.19 (1.42, 3.38) |  |
|  | 45 – 60 years | 370 | 14.8% | 43 | 11.6% | 4.28 (2.61, 7.03) |  |
|  | Over 60 years | 265 | 10.6% | 25 | 9.4% | 3.40 (1.93, 6.00) |  |
| Primary occupation | |  |  |  |  |  |  |
|  | Other occupation | 1965 | 78.5% | 118 | 6.0% |  | < 0.001 |
|  | Farmer/ plantation worker | 538 | 21.5% | 60 | 11.2% | 2.23 (1.52, 3.25) |  |
| Farm or plantation activities | | | | |  |  |  |
|  | No | 657 | 26.2% | 47 | 7.2% |  | 0.51 |
|  | Yes | 1846 | 73.8% | 131 | 7.1% | 1.17 (0.734, 1.86) |  |
| Stay overnight outside kampung | |  |  |  |  |  |  |
|  | No | 2116 | 88.4% | 161 | 7.6% |  | 0.02 |
|  | Yes | 387 | 11.6% | 17 | 4.4% | 0.53 (0.30, 0.95) |  |
| Elevation | | | | | | |  |
|  | Under 50 MSL | 1396 | 55.7% | 130 | 9.3% |  | < 0.001 |
|  | 50-100 MSL | 730 | 29.2% | 30 | 4.1% | 0.41 (0.25, 0.67) |  |
|  | Over 100 MSL | 377 | 15.1% | 18 | 4.7% | 0.46 (0.25, 0.86) |  |
| Forest within 50m of house | |  |  |  |  |  |  |
|  | Forest within 50m of house | 852 | 34.0% | 76 | 8.9% |  | 0.11 |
|  | Forest over 50m from house | 1651 | 66.0% | 102 | 6.2% | 0.71 (0.47, 1.07) |  |
| Proportion of forest within 1km of house | | | |  |  |  |  |
|  | Less than 30% | 421 | 16.8% | 17 | 4.0% |  | < 0.001 |
|  | 30-40% forest cover | 608 | 24.3% | 43 | 7.1% | 1.52 (1.04, 2.20) |  |
|  | 40 – 75% forest cover | 842 | 33.6% | 45 | 5.3% | 1.31 (0.67, 2.53) |  |
|  | Over 75% forest cover | 632 | 25.3% | 73 | 11.6% | 3.16 (1.64, 6.07) |  |
| Proportion of agriculture within 1km | |  |  |  |  |  |  |
|  | Less than 10% | 658 | 26.3% | 83 | 12.6% |  | < 0.001 |
|  | 10 – 25% agricultural land | 533 | 21.3% | 39 | 7.3% | 0.54 (0.33, 0.90) |  |
|  | 25 – 35% agricultural land | 656 | 26.2% | 35 | 5.3% | 0.37 (0.22, 0.62) |  |
|  | Over 35% agricultural land | 656 | 26.2% | 21 | 3.2% | 0.22 (0.12, 0.39) |  |
| Proportion of cleared/ open area within 500m of house | | | |  |  |  |  |
|  | Less than 15% | 530 | 21.2% | 40 | 7.5% | - | 0.01 |
|  | 15 – 30% cleared | 890 | 35.6% | 40 | 4.5% | 0.56 (0.32, 0.99) |  |
|  | 30 – 40% cleared | 579 | 23.1% | 49 | 8.5% | 1.16 (0.65, 2.05) |  |
|  | Over 40% cleared | 504 | 20.1% | 49 | 9.7% | 1.29 (0.72, 2.30) |  |

* Unadjusted odds ratios
